# Socioeconomic Associations with ADHD: Findings from a Mediation Analysis

**DOI:** 10.1371/journal.pone.0128248

**Published:** 2015-06-01

**Authors:** Abigail Emma Russell, Tamsin Ford, Ginny Russell

**Affiliations:** 1 Institute of Health Research, University of Exeter Medical School, Exeter, United Kingdom; 2 ESRC Centre for Genomics in Society (Egenis) & Institute of Health Research, University of Exeter Medical School, Exeter, United Kingdom; University of New South Wales, AUSTRALIA

## Abstract

**Background:**

Children from disadvantaged socioeconomic backgrounds are at greater risk of a range of negative outcomes throughout their life course than their peers; however the specific mechanisms by which socioeconomic status relates to different health outcomes in childhood are as yet unclear.

**Aims:**

The current study investigates the relationship between socioeconomic disadvantage in childhood and attention deficit/hyperactivity disorder (ADHD), and investigates putative mediators of this association in a longitudinal population-based birth cohort in the UK.

**Methods:**

Data from the Avon Longitudinal Study of Parents and Children was used (n = 8,132) to explore the relationship between different measures of socioeconomic status at birth-3 years and their association with a diagnosis of ADHD at age 7. A multiple mediation model was utilised to examine factors occurring between these ages that may mediate the association.

**Results:**

Financial difficulties, housing tenure, maternal age at birth of child and marital status were significantly associated with an outcome of ADHD, such that families either living in financial difficulty, living in council housing, with younger or single mothers’ were more likely to have a child with a research diagnosis of ADHD at age 7. Financial difficulties was the strongest predictor of ADHD (OR 2.23 95% CI 1.57-3.16). In the multiple mediation model, involvement in parenting at age 6 and presence of adversity at age 2-4 mediated 27.8% of the association.

**Conclusions:**

Socioeconomic disadvantage, conceptualised as reported difficulty in affording basic necessities (e.g. heating, food) has both direct and indirect impacts on a child’s risk of ADHD. Lower levels of parent involvement mediates this association, as does presence of adversity; with children exposed to adversity and those with less involved parents being at an increased risk of having ADHD. This study highlights the importance of home and environmental factors as small but important contributors toward the aetiology of ADHD.

## Introduction

Groups and individuals differ in societal position by the amount and type of resources held, be these economic, social or political [1]. Individuals and groups in differing socioeconomic strata are known to have disparate health outcomes, with those in the most disadvantaged groups at highest risk of poor health [2]. Children from disadvantaged socioeconomic backgrounds are at a greater risk of a range of negative outcomes throughout their life course compared with their peers [3], however the specific mechanisms by which socioeconomic status (SES) relates to different health outcomes in childhood are as yet unclear, perhaps due to the complex relationships between SES and health as well as individual patterns of resilience in each child. The current study investigates the relationship between socioeconomic disadvantage in childhood and one particular outcome: attention deficit/hyperactivity disorder (ADHD), and investigates putative mediators of this association in a longitudinal population-based birth cohort in the UK.

Low SES has been linked to poor health in childhood, specifically (but not limited to) an increased risk of dental caries [4], behavioural problems [5–7], increased risk of smoking initiation [8], slow growth/shorter stature [9], suboptimal cognitive development [3, 10, 11] and low birth weight [10]. Children from socioeconomically disadvantaged backgrounds are also more at risk of mental health problems [12]. In a systematic review of 55 studies that explored relationships between SES and childhood mental health outcomes 52 reported an inverse relationship between the two. Overall, children were 1.18–3.34 times more likely to have poor mental health if they were from socioeconomically disadvantaged backgrounds [12].

The current study focusses on associations between socioeconomic disadvantage and ADHD. ADHD is a psychiatric disorder with onset in childhood, which can persist throughout the life course [13]. ADHD is characterised by symptoms of hyperactivity, impulsivity and/or inattention that cause impairment for the individual across multiple settings [14]. It has been reported to have a prevalence in young people of 2–5% [15] and has a complex aetiology. Although the majority of risk is thought to be incurred through heritable factors-with data from 20 twin studies estimating heritability at around 0.76 [16], environmental and social influences are also likely to contribute to aetiology [17]. An individual with ADHD has an increased risk of a range of negative outcomes such as poor educational achievement and substance abuse [18], and this may interact with or exacerbate risks incurred through socioeconomic disadvantage. Although effective pharmacological and non-pharmacological treatments for ADHD exist, these target ADHD symptoms rather than causal processes [19]. Identification of social and environmental risk factors is an important alternate avenue for tackling this prevalent and impairing condition.

The association between socioeconomic disadvantage and ADHD appears to be complex and potentially mediated by other factors that may co-occur with low SES [20]. This may be because these other factors lie on a causal pathway between SES and ADHD, and therefore alter or account for this relationship (also known as mediation). Confounding may also play a role; socioeconomic status is measured in many ways and these are known to be inter-related, and many health-related behaviours occur differentially by an individual’s SES, for example those of lower SES are more likely to smoke [8, 21]. Furthermore, as ADHD and its associated traits are known to be highly heritable [16], parental low education as an SES indicator could in fact be confounded with the parent’s own ADHD traits, which led to their low educational attainment, and/or predisposition to smoke [22]. The same traits could also lead to a parent having a lower occupational status due to their preference for hands-on or active work, which has been classed as socioeconomically lower than other occupations. The child being diagnosed with ADHD may therefore reflect inherited genetic traits rather than ADHD being caused by their parents’ low SES. An alternate hypothesis is that having a child with ADHD causes socioeconomic disadvantage within the family. One study found that lower labour supply and increased risk of relationship instability in parents of children with ADHD was only half accounted for by socioeconomic disadvantage, and conclude that having a child with ADHD reduces parental SES [23], although others have found little or no support for such theories of reverse causality [20].

An alternate explanation for the association between SES and ADHD is passive gene-environment correlation, whereby the environment and the genes provided to children by their parents may themselves be correlated [24]. For example the home environment, parenting behaviours and the socioeconomic standing of parents are all potentially influenced by their ADHD genotype. Their children then inherit this genotype which will influence their own developmental and socioeconomic pathways [25].

Existing literature suggests a strong association between ADHD and SES, and possible mediators in the family and home environment as these have been highlighted as potential mechanisms to explain the association [20, 26]. Putative mechanistic factors that have been proposed include maternal mental health [26], substance abuse [27] and aspects of the home environment [26].

Parental depression is known to negatively affect child outcomes [28], and parental substance abuse or other psychopathology can also impact negatively on the parent-child relationship [29]. Parental depression and anxiety has been associated with attention problems in young children, which may be due to negative impacts on parenting and parent-child attachment [30]. Others have found that the negative association between income and child health has been almost entirely accounted for by mother’s mental-health [31]. The level of involvement that a parent has with their child’s upbringing impacts upon the way that self-regulatory mechanisms develop [32], which theoretically could underlie the symptoms of ADHD [33]. Parents who are highly involved in a child’s upbringing may promote joint attention and self-regulation. Fathers’ with higher incomes report more involvement with their child [34]. Childhood diet (specifically increased additives such as preservatives and colouring) may increase hyperactivity in children [35]. Family adversity such as partner cruelty, substance abuse and parental criminal involvement are considered risk factors for various forms of psychopathology including ADHD [36, 37]. Research using indices of adversity [38] has found it is the number of risk factors (and their cumulative effects) rather than the specific risk which is of importance.

Using a large, population—based birth cohort from the UK, our objectives for the current study were to:-

- Assess if there are there individual-level associations between parental income, occupation, education and single-parent status and ADHD in the child
- Establish which of these socioeconomic associations with ADHD is strongest
- Examine proximal home and family factors such as parent mental health, parenting involvement and psychosocial adversity as potential mediators of this effect

We hypothesised that indicators of SES would be independently negatively associated with an outcome of ADHD, and that parental education would be the strongest predictor of this association due to genetic confounding (i.e. parents of children with ADHD will themselves tend to have the difficulties with attention, hyperactivity and impulsivity associated with ADHD and therefore, be more likely to have poor educational attainment). We also hypothesised that the association between ADHD and SES would be mediated in part by family and home environmental factors such as parental psychopathology, family adversity (e.g. presence of domestic violence, substance abuse) and parenting involvement.

## Methods

### Design and Participants

This study utilises longitudinal data from the Avon Longitudinal Study of Parents and Children (ALSPAC) birth cohort. Full details of the methodology and profiles of the cohort are published elsewhere [39–41]. In brief, all pregnant women living in a defined geographical area (Avon) in South-West England with an estimated delivery date between 1^st^ April 1991 and 31^st^ December 1992 were initially invited to enrol in the study, with supplementary recruitment taking place in two further phases. Of the 15,458 foetuses, 14,775 were live births and 14,701 were alive at one year of age.

Mothers, their partners [the term ‘partner’ will be used henceforth to encompass both fathers’ and partners’ of the mother who are not the study child’s biological parent] and the study child have been followed up by a combination of questionnaires, clinic visits and assessments [41]. Sample size was limited to children whose parents completed the Development and Wellbeing Assessment (DAWBA), a standardised diagnostic measure used in the current study to assign research diagnoses of ADHD at age 7. ALSPAC collected data at every time point for both twins when there was a twin birth but excluded triplets and quadruplets from the cohort. For the purpose of this study, one twin in each pair was randomly deleted as ADHD is commonly concordant in twins [16]. These criteria resulted in an overall sample size for the current study of 8,132 children and their parents/carers.

### Measures

#### Socioeconomic Status

SES was measured in eight ways in order to test the relative predictive abilities of different indicators. The exact wording of the questions that parents responded to can be seen in S1 Table. [42]

Parental income: Self-reported family income (mother report) was measured when the study child was 33 months old. This was reported in five increments of £100, from less than £100 per week, to £400 and over. A binary measure ‘financial difficulties’ was also included, when the parent reported difficulty in affording heating, clothing, rent/ mortgage, food and/or things for the study child [43].

Parent Education: Mother and partner education levels were classified as less than GCSE, GCSE (or equivalent), and higher than GCSE. GCSE’s are the UKs standard exams at age 16, and mark the end of mandatory schooling. The educational attainment of mothers was recorded at 32 weeks gestation; however data on partners’ education level was not available until the child was aged eight. It is unlikely however, that many partners’ education substantially changed during that time, as most of the study sample will have completed their education prior to having children.

Parent Employment: Employment of mothers and partners (as reported by mothers) was recorded 32 weeks into the pregnancy and was classified into four categories; unemployed; housewife/husband or retired; in education/training and employed.

Marital Status/Family structure: Mothers provided information at 8–12 weeks gestation about their family structure which was classified into single/ cohabiting /married.

Maternal age at birth of study child: Mothers’ age in years at the birth of the study child was recorded.

Housing Tenure: Mothers reported on their housing status at 8–12 weeks gestation. This was divided into renting through the council/housing association (social housing); private renting and home owner.

Large family size: Mothers who had reported living with more than three biological children or more than two other children during the period where the study child was aged 0–2 was classed as large family size. Having a large family is known to put pressure on a household’s economic resources [44] and so was included as a socioeconomic measure in the current study.

#### Outcome measure- ADHD age 7

The DAWBA was used to evaluate psychological disorders in the study children at 7 years 7 months old. It is a validated instrument combining structured and semi-structured questions related to DSM and ICD diagnostic criteria [45]. There are parallel versions for different informants; in ALSPAC, DAWBA data was collected via questionnaires posted to parents and teachers. Responses from both informants were reviewed by trained clinical raters who assigned diagnoses according to the DSM-IV [46]. Clinical raters reviewed both structured and qualitative information from all available informants (parents and teachers). These were combined to assign a diagnosis as would occur in a clinical setting. The inter-rater reliability for the DAWBA is substantial with kappas of approximately 0.75 for different disorders among samples with an IQ >70 [47]. Detailed investigation with both community and clinical samples has demonstrated its validity [45]. Using the DAWBA, 175 children were assigned a research diagnosis of ADHD at this time point, comprising 2.13% of the study sample.

#### Mediators

When exploring aetiological theories or mediational models, the use of longitudinal as opposed to cross-sectional data are important, as researchers can ensure that the exposure is measured before the mediator and the mediator is measured prior to the outcome [48]. Due to ongoing data collection throughout the child’s life, family and home-based mediators were chosen that had occurred (or impacted) on the child between birth and age seven. This allows a model that occurs across time; SES at birth may be mediated by factors throughout early childhood leading to a diagnosis of ADHD.

Parental psychopathology: Mother and partners were classed as being depressed if they had a score of 13 or more on the Edinburgh Postnatal Depression Scale, a scale validated for use both during and outside of pregnancy [49]. Data was collected from mothers when the child was 2 years 9 months old and in partners when the child was 1 year 9 months.

Parenting activities age 6: Mothers were asked in detail when the child was aged 6 years 9 months about activities herself and her partner engaged in with the child. This gave a total score out of 75 for each parent, with higher scores indicating more involvement in activities with the child.

Fizzy drinks/caffeine consumption at 3 years old: Parents were asked to report on how often their child drank cola and fizzy drinks, which was aggregated to form a variable for each time point of “never” “less than once a week” and “more than once a week”, based on reports of the frequency the child was drinking fizzy drinks or cola.

Family adversity age 2–4: The family adversity index (FAI) [43] is an index developed in ALSPAC based on Rutter’s original indicators of adversity [50] and records family-based risk factors. The presence of at least one of the following factors was considered to indicate exposure to adversity in the current study; lack of partner affection; partner cruelty (considered present if the mother had reported she had been hurt by her partner physically or experienced emotional cruelty from her partner); family major problems; psychopathology of mother, substance abuse (this included use of “hard” drugs or alcohol consumption of more than three glasses a day for more than ten days) and crime (trouble with the police). In addition to this dichotomised indicator, partner cruelty and substance abuse were investigated as putative mediators.

#### Analysis

Continuous variables were checked to ensure that they were normally distributed. For ease of interpretation, scores were reversed so for all the mediators an increase in score represented a more negative impact (e.g. more fizzy drinks, less parental involvement). Descriptive statistics detailed differences in means/frequencies between those with an outcome of ADHD and those without for the predictors and mediators. Unadjusted logistic regression was carried out between each SES predictor and ADHD outcome. Multivariable regression was then used with those significant predictors to derive an SES model that explained the largest possible variance in the outcome.

The predictor with the strongest relationship to the outcome was then used in a mediation model. Multiple mediation analysis was carried out as recommended by Preacher and Hayes [51] using the products of coefficients approach. Candidate mediators that showed significant associations with both the predictor and the outcome were included in the final mediation model, which was adjusted for gender. Bootstrapping was used in order to estimate bias-corrected confidence intervals. Mediation analysis was carried out with the commands “*binary_mediation* “and “*bootstrap”* in Stata 13 [52].

To assess the effect of missing data, descriptive statistics were reported to examine differences in the predictors between the entire ALSPAC cohort and the study sample (a subsample who completed the DAWBA assessment at age 7). These are shown in S2 Table. Between 66% and 93% of the eligible sample did not respond to individual questionnaires, thus some data were missing from analysis. These data were not missing at random, as low SES itself predicted drop-out [53]. Multiple imputation was therefore conducted and used for the analyses with the exception of the mediation model, where the statistical commands were incompatible. We imputed based on the SES variables and birth weight, gender and gestation using the *mi impute* command in Stata 13.

#### Ethical approval

Ethical approval for the study was obtained from the ALSPAC Ethics and Law Committee and the Local Research Ethics Committees. The University of Exeter Medical School Research and Ethics Committee also provided approval for the current study.

## Results

Mothers of children with ADHD had slightly lower levels of education, and proportionately more of the ADHD group had incomes within the lowest two bands (see Table 1). The ADHD group had proportionately more participants in the lower housing bands: council/housing association housing (17.8% in the ADHD group vs 10.1% in the no diagnosis group). Mothers with children with ADHD were less likely to be married than mothers of children with no ADHD diagnosis; 72.6% compared with 81.2% respectively. Proportionately more of the families of children with ADHD reported being in financial difficulty (27.78% vs 14.44% respectively) or having a large family (7.74% vs 4.87% respectively). There was a larger proportion of boys in the ADHD group (83.9% vs 50.5% in the no ADHD diagnosis group), and more mothers reported smoking during pregnancy in the ADHD group (26.6% vs 19.2% of the no ADHD diagnosis group).

**Table 1:** Descriptive statistics; families with children diagnosed with ADHD at age 7 compared with those with no ADHD diagnosis.

	Diagnosis of ADHD		No Diagnosis of ADHD	
Predictors				
Weekly Income (%)	*n = 136*		*n = 6*,*562*	
<£100		9.56		6.99
£100-£199		21.32		15.79
£200-£299		27.21		28.6
£300-£399		17.65		22.68
>£400		24.26		25.94
Education of mother (%)	*n = 162*		*n = 7*,*706*	
< GCSE		24.69		23.28
GCSE		38.27		35.08
>GCSE		37.04		41.64
Education of partner (%)	*n = 109*		*n = 5*,*694*	
<GCSE		8.26		4.79
GCSE		50.46		47.00
>GCSE		41.28		48.21
Housing tenure (%)	*n = 157*		*n = 7*,*521*	
Council/HA rent		17.83		10.09
Private rent		5.10		5.80
Own/mortgage		77.07		84.11
Marital Status (%)	*n = 164*		*n = 7*,*775*	
Single		14.63		9.27
Cohabiting		12.80		9.50
Married		72.56		81.22
Employment- mother (%)	*n = 136*		*n = 6*,*621*	
Unemployed		4.41		3.38
Housewife/retired/education		44.85		46.05
Employed		50.74		50.57
Employment- partner (%)	*n = 151*		*n = 7*,*352*	
Unemployed		7.95		6.24
Househusband/retired/education		1.99		2.16
Employed		90.07		91.59
Mothers age at birth, years, mean (SD)	*n = 172*	28.11 (4.97)	*n = 7*,*933*	28.97 (4.60)
Large family size (% with)	*n = 168*	7.74	*n = 7*,*757*	4.87
Financial difficulties (% with)	*n = 162*	27.78	*n = 7*,*720*	14.44
**Covariates**				
Gestation in weeks, mean (SD)	*n = 172*	39.08 (2.34)	*n = 7*,*933*	39.49 (1.81)
Male child (%)	*n = 174*	83.91	*n = 7*,*958*	50.53
Birth weight in g, mean (SD)	*n = 172*	3390.12 (600.38)	*n = 7*,*841*	3431.11 (535.86)

Note: number of observations- not all participants recorded data for every characteristic. Missing data were excluded from the analysis. HA = housing association, GCSE = General Certificate of Secondary Education.

As shown in Table 2, in unadjusted logistic regression significant predictors of ADHD were housing tenure, marital status, mothers’ age at birth and financial difficulties, such that a child is more likely to have an outcome of ADHD if either they lived with a single parent (OR 1.70 95% CI 1.09–2.66) or their family lived in a council/housing association property (OR 1.84 95% CI 1.22–2.76). Children were marginally less likely to receive a diagnosis of ADHD if their mother was older when the child was born (OR 0.96 95%CI 0.93–0.99). Children were over twice as likely to have ADHD if their family was in perceived financial difficulty when they were an infant (OR 2.23 95%CI 1.57–3.16).

**Table 2:** Unadjusted logistic regression of each socioeconomic predictor on the outcome (ADHD diagnosis at age 7).

Predictors	OR (95% CI)	*p*
Weekly Income (%)	N = 8,132	0.074
>£400	Reference	
£300-£399	0.88 (0.52–1.48)	
£200-£299	1.12 (0.71–1.77)	
£100-£199	1.61 (1.00–2.60)	
<£100	1.72 (0.94–3.15)	
Education of mother (%)	N = 8,132	0.406
> GCSE	Reference	
GCSE	1.25 (0.88–1.79)	
<GCSE	1.23 (0.83–1.83)	
Education of partner (%)	N = 8,132	0.067
>GCSE	Reference	
GCSE	1.34 (0.93–1.92)	
<GCSE	2.01 (1.09–3.71)	
Housing tenure (%)	N = 8,132	0.014
Own/mortgage	Reference	
Private rent	0.97 (0.48–1.97)	
Council/HA rent	1.84 (1.22–2.76)	
Marital Status (%)	N = 8,132	0.029
Married	Reference	
Cohabiting	1.48 (0.92–2.40)	
Single	1.70 (1.09–2.66)	
Employment- mother (%)	N = 8,132	0.847
Employed	Reference	
Housewife/retired/education	0.99 (0.71–1.39)	
Unemployed	1.26 (0.56–2.84)	
Employment- partner (%)	N = 8,132	0.610
Employed	Reference	
Househusband/retired/education	1.01 (0.33–3.10)	
Unemployed	1.34 (0.74–2.43)	
Mothers age at birth, years, mean (SD)	N = 8,132	
	0.96 (0.93–0.99)	0.017
Large family size	N = 8,132	
	1.59 (0.89–2.82)	0.115
Financial difficulties	N = 8,132	
	2.23 (1.57–3.16)	<0.001

Note: OR = odds ratio CI = confidence interval HA = housing association, GCSE = General Certificate of Secondary Education.

### What is the strongest predictor of ADHD (stepwise regression)

A multivariable regression model (see Table 3) using significant individual SES predictors of ADHD (financial difficulties, housing tenure, marital status and maternal age) was used to explore which predictors of ADHD explained the most variance. Financial difficulties was the only predictor which remained significant in the presence of the other significant SES indicators, therefore it was used in as the predictor in the mediation analysis.

**Table 3:** Multivariable regression with socioeconomic predictors which were significant in unadjusted logistic regression model on the outcome (ADHD diagnosis at age 7).

Variable	OR	95% CI	p value
Financial Difficulties	2.06	1.44–2.94	<0.001
Housing tenure			
*Own/mortgage*	*Ref*		
*Rent*	0.72	0.35–1.51	0.386
*Council/HA*	1.30	0.82–2.05	0.265
Marital Status			
*Married*	*Ref*		
*Cohabiting*	1.28	0.77–2.12	0.347
*Single*	1.35	0.83–2.18	0.224
Maternal age	0.97	0.94–1.01	0.138

### What are the mechanisms for this effect? (Mediation)

Parent report of financial difficulties was regressed on ADHD with adjustment for gender.

**Fig 1:**
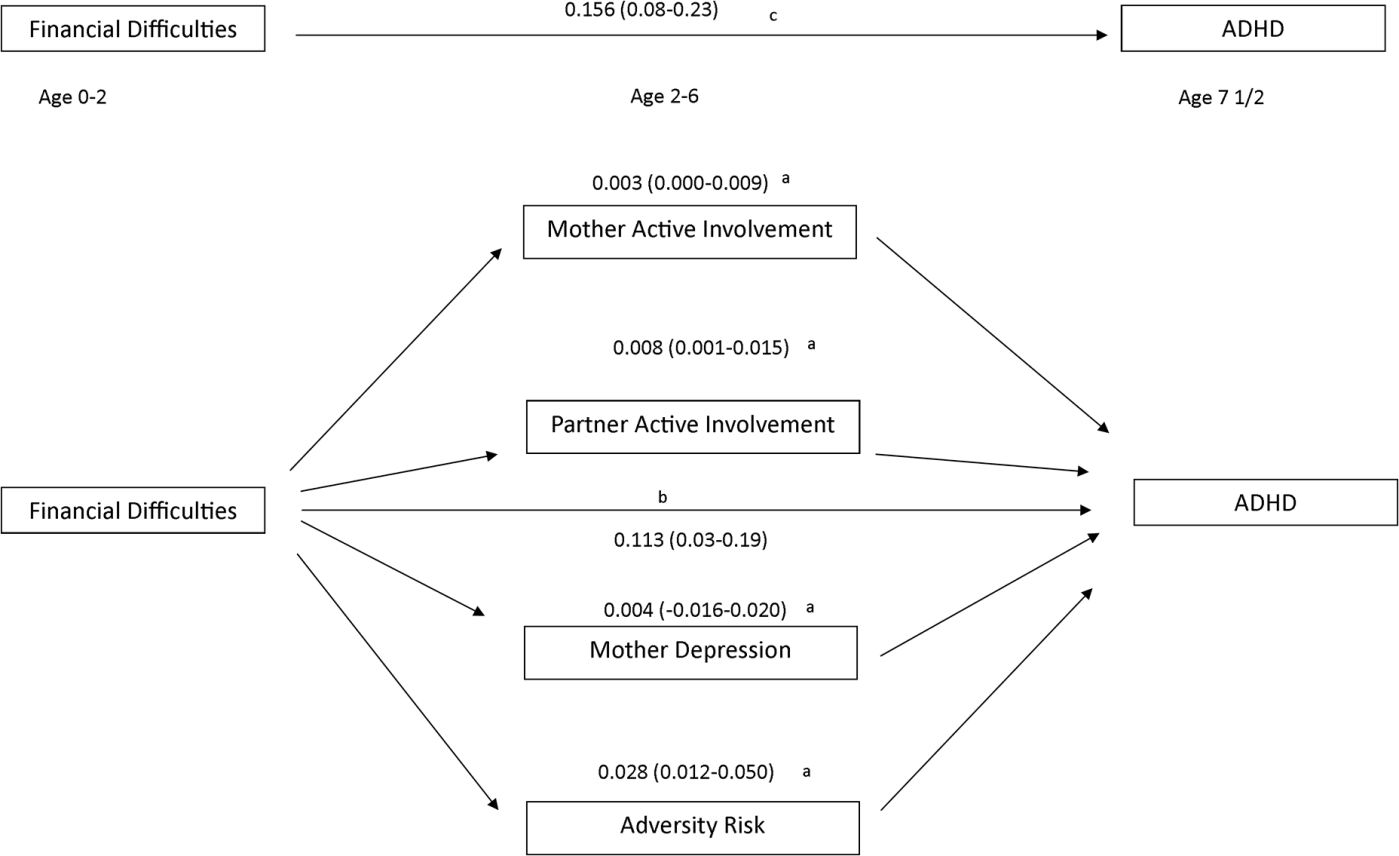
Mediation model. Notes: a- indirect effects b- direct effects c- total effect. Numbers refer to coefficient (bias-corrected 95% confidence interval) for each path

Four mediators were then tested in one model with financial difficulties at age 0–2 as the predictor and ADHD diagnosis at age 7 as the outcome. These were mother and partner involvement with the study child, maternal depression and presence of family adversity. In the multiple mediation model, bias corrected confidence intervals excluded zero (representing statistical significance at the 5% level) for the direct effect (see Fig 1 (C) and Table 4), the total effect (b) and for three mediators (a). There was evidence that lower levels of parental involvement, both of the mother and partner and presence of family adversity mediated the link between financial difficulty and ADHD. Mothers’ depression at 33 months was not a significant mediator in this model. The relative strength of each mediator can be seen in Fig 1. Overall, 27.8% of the total effect between financial difficulties at age 0–2 and ADHD at age 7 was mediated, with the majority of mediation occurring through adversity risk at age 2–4 (coefficient 0.03, bias-corrected (BC) CIs 0.01–0.05) while lower levels of both mother and partner involvement at age 6 also mediated the association (mother coefficient 0.003 BC CI’s 0.000–0.009; partner coefficient 0.008, BC CI 0.002–0.016).

**Table 4:** Mediation analysis.

Path	Coefficient	Bias corrected 95% Confidence Intervals
Total effect	0.156	0.082–0.228
Direct Effect	0.113	0.034–0.189
Indirect Effects:		
Adversity Risk	0.028	0.012–0.050
Mother involvement	0.003	0.000–0.009
Partner involvement	0.008	0.002–0.016
Mother depression	0.004	-0.012–0.020

## Discussion

Our findings confirmed there were associations between some indicators of socio-economic disadvantage, namely financial difficulties, social housing tenure, younger maternal age, single-parent status and ADHD in the child. Although ADHD was more prevalent in families whose parents had lower occupational status, and lower educational levels, these indicators did not show significant association with ADHD in children in the current sample. Financial difficulty, conceptualised as reported difficulty in affording heating, clothing, rent/ mortgage, food and/or things for the study child, was the socioeconomic indicator which was associated with the greatest increased odds for ADHD. Those whose mothers were classed as in financial difficulty when the child was aged 0–2 were 2.23 times more likely to have a research diagnosis of ADHD at age 7 than their peers. This association was mediated by how involved both parents were with their child and by the presence of family adversity, such that children with less involved parents and with at least one type of family adversity were more likely to receive a diagnosis of ADHD.

Although we hypothesised that parental education would be the strongest predictor of ADHD, this was not the case. The reasoning behind this hypothesis was that gene-environment correlation was likely to be high for ADHD and educational attainment, therefore those who did not attain highly in education would be more likely to have ADHD-like traits and pass these genes on to their children. Instead, financial difficulties emerged as the strongest predictor. Financial difficulties may be an indicator of very severe deprivation, which may be alter the aetiology of ADHD whereas more common, albeit disadvantageous, family circumstances did not emerge as a strong risk factor.

A substantial proportion of the relationship between financial difficulties and ADHD was mediated by adverse family factors and adversity risk (28% of the relationship was mediated by the factors tested). This lends weight to theory that parent involvement mediates the relationship between SES and ADHD. Severe financial difficulties may co-exist with extremely under-resourced parenting, in some cases leading to lack of parent involvement. There are numerous studies that show ADHD is more common in extremely challenging home environments: for example among children who are looked after, neglected or who have been abused [54, 55]. The finding that many aspects of home life can mediate the pathway to ADHD supports models that suggest a number of risk factors (and their cumulative effects) may be important [38].

Webb [56] suggests that epigenetic mechanisms may underpin the association observed. She argues that family environments of profound neglect may lead to alterations in gene expression as a result of DNA methylation, and convincingly demonstrates there is a socio-economic gradient associated with child abuse and child neglect. Our findings suggest severe adversity was the biggest single mediator linking low SES to ADHD. This meant the presence of major family problems, family psychopathology, family substance abuse, physical violence or crime could engender hyperactivity and inattentive behaviours in children. Our finding does suggest that it is these very extreme family circumstances that are likely to elicit ADHD-type behaviours.

SES has been defined as “a broad concept that refers to the placement of persons, families, with respect to the capacity to create or consume goods that are valued in our society” [57]. The breadth of SES as a concept means there are many potential ways to conceptualise and measure SES, both at the individual and geographical/group level [58]. Common individual-level SES measures of in children are parental educational level, occupation, income, marital status (or number of adults in the household), maternal age at the child’s birth and/or housing tenure [59]. The SES measures in ALSPAC were, as expected, inter-related. Logistic regression of the socioeconomic predictors on ADHD showed that a mothers’ report of whether the family struggled to afford goods is a more accurate predictor of ADHD than their actual weekly income or other socioeconomic measures. The measure of financial difficulties asks about being able to afford goods that may directly impact on health; inadequate food, clothing and housing may all contribute to poor child health and impaired development. Results from the mediation analysis suggests that there are both direct effects of financial difficulties on ADHD as well as mediation by parental involvement and adversity risk, and that the mediators in total still do not have as much impact on the outcome as the direct effect.

Our findings concur with recent research that found that home learning environment, which included aspects such as reading to children (overlapping with current study measure of parent involvement), mediated the relationship between SES and ADHD [60]. With regard to paternal involvement, a recent study reported that for fathers with ADHD symptoms, an association with conduct problems in the child (also with ADHD) were only found when the father also had high levels of involvement in childrearing [34]. In addition, the authors found that those fathers with higher incomes reported higher levels of involvement with their child. This complex relationship needs to be further investigated to disentangle the directions of effects in order to best target intervention, as there may be different impacts of father involvement depending on their own ADHD symptoms as well as their socioeconomic circumstances.

Being a single parent has been associated with an increased risk of ADHD for the child both in the current study and previously [23, 27, 61]. There are various mechanisms through which single parenthood links with other measures of SES and through which this may influence a diagnosis of ADHD. For example, one parent must earn or bring in an income as well as raise any children. In addition a lone parent may experience increased stress, and as a result be more likely to use suboptimal parenting strategies[30]. Mental illness such as depression may precede or follow, further compounding the difficulties of effective parenting. As the majority of those diagnosed with ADHD in the current study were boys, the association with single parenthood may indicate that young boys lacking a male role model are more susceptible to the disorder, which is supported by our finding that partner involvement acts as a mediator between SES and ADHD. However, being a single mother may be positive for a family if she has left an abusive or unhealthy relationship [62].

It is of interest that having married parents decreases the risk of ADHD more so than does having cohabiting parents. In the Millennium Cohort Study, single parent families have substantially lower household incomes than cohabiting parents, and married parents have a higher income than cohabiting parents, reflecting an interaction between marital status and other aspects of SES [63]. There is little difference in child outcomes by marital status of the parents after controlling for background characteristics that indicate selection into marriage (e.g. relationship quality and parental cognitive ability), and other socioeconomic factors (parent education and income) [64] which implies that the characteristics of those who chose to marry, rather than marriage itself, act as a protective factor for child outcomes.

In the current sample, ADHD is more prevalent among the children of younger mothers, although this association is small. Mothers’ age again ties in with socioeconomic status; as wealth and resources accumulate over time, younger mothers are often financially worse off than their older counterparts, and their education may have been interrupted by their pregnancy. Being a younger mother may also represent an increased likelihood of the study child being the first-born and unplanned, and this could manifest in differences in parenting experience and management of disruptive behaviour between younger and older mothers with single or multiple children. If the child was unplanned this may also reflect on the mother’s own ADHD-like tendencies; poor planning and risk taking behaviour, known facets of ADHD, may result in unplanned pregnancy.

We found that 27.8% of the direct effect of financial difficulty was mediated. This implies that there is a direct consequence of a family suffering financial difficulty on aspects of parenting and the family/home environment that exacerbate expression of a child’s ADHD symptoms. Such family stressors could compound hyperactive behaviour. It may also be that other aspects of the home environment which lie on this pathway that were not investigated in the current study further mediate the SES-ADHD association, for example through disorganised attachment patterns [65].

### Limitations

The current study has many strengths; a large, representative longitudinal birth cohort allowed for modelling of causal processes that may occur throughout childhood. ADHD was measured in the whole population, as opposed to the selection biases inherent in a clinically referred sample, giving a much clearer picture of underlying risk. Multiple imputation was used to address the limitation of missing data in the first parts of the analysis, however the binary mediation with bootstrapping and multiple imputation commands were mutually incompatible, therefore the mediation analysis was carried out with the original data. Logistic regression using the original (missing cases omitted) data are shown in S3 Table. The results were very similar to those with the imputed data; therefore we considered the mediation model which utilised the original data to be robust.

As discussed by Wolke, [53] due to the systematic drop-out in ALSPAC of those of lower SES, conclusions drawn from the utilising the original data and not the imputed data in the mediation analysis are likely to underplay the effects found in this study as compared with the original ALSPAC cohort. The power of our analysis was limited by the relatively small number of children with ADHD in the study sample, and sadly there was no measure of parental ADHD or traits, which meant that we could not estimate whether genetic confounding and interaction between parent ADHD and their behaviours (such as in the case of father involvement [34]) could not be investigated.

### Future Research

Future studies using a sample exposed to a wider variety of adversities (such as the E-Risk study [66]) may be able to determine if there is a dose-response relationship between adversity exposure and ADHD symptoms. Future research could further attempt to unpack the effects of specific aspects of socioeconomic disadvantage on ADHD as well as other child mental health problems. Replication using a genetically informed study design or accounting for parental psychopathology, in particular ADHD, would add weight to the current findings.

Our results raise the question of whether, and to what extent the development of ADHD is influenced by the social, and specifically home and family context. Although relative effects of socioeconomic, home and family factors are likely to be small, they are important because unlike genetic predisposition or genetic risk, they can be current targets for intervention. The results also underline that the notion of ADHD as an entirely fixed underlying biological entity requires qualification, as noted elsewhere [67].

Clinicians should be aware that children and young people presenting with symptoms of ADHD are likely to have complex and often difficult family circumstances. Taking a holistic approach to treatment and referral on to other services that may support the families’ to cope with their socioeconomic situation are important aspects of care for these children. On a societal level the results of this study question whether the current benefits system in the UK provides sufficient support for families with children to afford basic necessities such as food, heating and clothing, which are necessary in order to promote health and wellbeing. Cross-cultural research exploring the prevalence of ADHD in societies with differing social support systems may further elicit the impact that inadequate living conditions has on rates of ADHD.

## Supporting Information

S1 Table Exact question wording for study measures. (DOCX)

S2 Table Descriptive statistics by entire ALSPAC cohort (N = 15,243) and study sample (N = 8,132). (DOCX)

S3 Table Results of logistic regression and multivariable regression with original and imputed data. (DOCX)
