# Supplementary material for: Socioeconomic Associations with ADHD: Findings from a Mediation Analysis
Source: PLoS One. 2015 Jun 1;10(6):e0128248. doi: 10.1371/journal.pone.0128248 (PMC4451079; doi:10.1371/journal.pone.0128248)
Supplement: S1 Table — (DOCX) [file pone.0128248.s001.docx]

Supporting information S1: **Exact question wording for study measures**

| **Measure** | **Exact wording of question and use in current study** | **Possible Responses** | **Respondent** | **Age of child** |
| --- | --- | --- | --- | --- |
| **Predictors** |  |  |  |  |
| Income | On average, about how much is the take home family income each week (include social benefits etc)? | Less than £100; £100-£199; £200-£299, £300-£399, £400 or more, don’t know | Mother | 33 months |
| Education | What educational qualifications do you, your husband or partner, your mother, and your father have? Please tick all that apply. (By husband or partner we mean your current live-in husband or partner).  *Note: results categorised into <GCSE, GCSE or >GCSE based on highest educational level reported for mother and partner* | CSE or GCSE (D, E, F or G); O-level or GCSE (A, B, or C); A-level; d) Qualifications in shorthand/  typing/or other skills, e.g. hairdressing; Apprenticeship;  State enrolled nurse; State registered nurse; City & Guilds intermediate  Technical; City & Guilds final technical; City & Guilds full technical; Teaching qualification; University degree; No qualifications; Qualifications not known; Not applicable, no such person; Other | Mother  Mother (for partner) | 32 weeks gestation  97 months |
| Employment | What is the present employment situation of yourself and your partner? Please tick all that apply.  *Note: categorised into unemployed; housewife/husband or in education or training or retired; employed.* | Working for an employer full-time (more than 30 hours a week); Working for an employer part-time (one hour or more a week); Self-employed, employing other people; Self-employed, not employing other people; On a government employment or training scheme; Waiting to start a job already accepted; Unemployed and looking for a job; At school or in other full-time education; Unable to work because of long-term sickness or disability; Retired from paid work; Looking after the home or family; Other (please describe) | Mother | 32 weeks gestation |
| Marital Status | Derived into single, cohabiting or married from two variables: What is your present marital status?  Please indicate who the adults over 18 in your household are | Never married; widowed; divorced; separated; married (once only); married for second or third time  Yourself; your partner; your parent(s); your partner’s parent(s); other relations of yours; other relations of your partner; friend(s); lodger; other | Mother | 8 weeks gestation |
| Housing Tenure | Is your home…?  *Note: categorised into own/mortgage, private rent and council or housing association rent* | being bought/mortgaged; owned - with no mortgage to pay; rented from council; rented from private landlord – furnished; rented from private landlord – unfurnished; rented from housing association; other (please describe) | Mother | 8 weeks gestation |
| Financial Difficulties | Derived from questions asking “How difficult at the moment do you find it to afford these items? Food, clothing, heating, rent or mortgage, things you need for the baby.  *Note: scores were from 0-15 with 15 representing high financial difficulty. The cut-off used in the current study was >8* | For each item: very difficult; fairly difficult; some difficulty; not difficult. | Mother | 8 months and 21 months |
| Large family size | Calculated as >3 children and >2 other children  We are interested in the other children who live with your baby. Please include half-brothers and half-sisters, step-brothers and step-sisters, fostered or adopted children. Do any other children live with you? How many boys/girls?  How many people live in your household now? (including yourself) | Number of other children  Number of children (under 16 years) | Mother  Mother | 6 months  21 months |
| **Covariates** |  |  |  |  |
| Mothers age at birth | Supplied by ALSPAC |  |  |  |
| Gender of child | Supplied by ALSPAC |  |  |  |
| Birth weight | Supplied by ALSPAC |  |  |  |
| Smoking during pregnancy | Did you smoke regularly at any of the following times in the last 9 months?  *Note: dichotomised into smoked during the first trimester or not* | Before pregnancy; first 3 months of pregnancy; last 2 weeks | Mother | 18 weeks gestation |
| Gestation | Supplied by ALSPAC |  |  |  |
| **Putative Mediators** |  |  |  |  |
| Parental Depression | Edinburgh Postnatal Scale depression score of 13 or higher |  | Mother  Partner | 33 months  21 months |
| Parenting activities | Frequency of involvement of a mother or father figure with the study child on 19 everyday activities e.g. helping the child get ready for school, reading to the child, preparing food for the child  *Note: used as a continuous score with higher scores representing less parental involvement* | For each item: nearly every day; 2-5 times a week; once a week; less than once a week; never | Mother | 81 months |
| Adversity Present | At least one risk present on the 2-4 years of age family adversity index (for more information see Wolke, 2004), with those used as predictors in this study (family size and financial difficulties) removed | Risks include: partner affection (lack of), partner cruelty, family major problems, maternal psychopathology, substance abuse and crime trouble with the police | Mother | 0-2 years |
| Substance abuse | Derived from several variables at two time points including mother consumed ‘hard drugs’ since the child was 18 months old and mother and partner high levels of alcohol consumption. Either alcohol or drug abuse had to be present to be considered ‘substance abuse’ Since your study child was 18 months old have you taken the following? Heroin, methadone, crack, cocaine  How much alcohol do you drink?  Which of the following statements about alcohol best apply to your partner?  If the mother reported drinking more than three glasses of wine a day for more than ten days or if she reported that her partner drank the same amount or more every day this was considered substance abuse. | Every day, often, sometimes, not at all  Never drink alcohol, very occasionally (< once per week), occasionally (at least once a week), drink 1-2 glasses nearly every day, drink 3-9 glasses every day, drink at least 10 glasses a day (glass defined as half a pint of beer or a glass or wine) | Mother | 33 months and 47 months |
| Partner cruelty | Derived from two questions repeated at two time points, present if the mother indicated she had been affected:  Listed below are a number of events which may have brought changes in your life. Have any of these occurred **since the study child was 18 months old?** If so, please assess how much effect it had on you….Your partner was physically cruel to you?...Your partner was emotionally cruel to you?...  If the mother reported she had been in any way “affected” by her partner being physically cruel or emotionally cruel, this was considered to indicate that ‘partner cruelty’ was present | Yes and affected me a lot; yes, moderately affected; yes, mildly affected; yes but did not affect me; no, did no | Mother | 33 months and 47 months |
| Fizzy drinks | Based on the two below questions children were categorised as having fizzy drinks never, less than once a week or more than once a week.  How many times in a week nowadays does [study child] drink…Cola drinks e.g. cola, pepsi?....other fizzy drinks e.g. lemonade?.... | Never, once in two weeks, 1-3 times a week, 4-7 times a week, > once per day | Mother | 38 months |
| **Outcome** |  |  |  |  |
| ADHD | Clinician diagnosis of any type of ADHD based on the DAWBA, see Goodman et al. 2000 [1] |  | Mother, teacher, clinician | 91 months |

References

1. Goodman, R., et al., The Development and Well-Being Assessment: description and initial validation of an integrated assessment of child and adolescent psychopathology. Journal of child psychology and psychiatry, 2000. **41**(05): p. 645-655.
