# Supplementary material for: Socioeconomic Associations with ADHD: Findings from a Mediation Analysis
Source: PLoS One. 2015 Jun 1;10(6):e0128248. doi: 10.1371/journal.pone.0128248 (PMC4451079; doi:10.1371/journal.pone.0128248)
Supplement: S2 Table — (DOCX) [file pone.0128248.s002.docx]

Supporting information S2: **Descriptive statistics by entire ALSPAC cohort (N=15,243) and study sample (N=8,132)**

|  | **ALSPAC Sample** | **Current Study Sample** |
| --- | --- | --- |
| Weekly Income (%) | *n=8,735* | *n=6,698* |
| <£100 | 8.76 | 7.05 |
| £100-£199 | 17.65 | 15.90 |
| £200-£299 | 28.39 | 28.58 |
| £300-£399 | 21.19 | 22.57 |
| >£400 | 24.01 | 25.90 |
| Education of mother (%) | *n=12,338* | *n=7,868* |
| < GCSE | 30.06 | 23.31 |
| GCSE | 34.62 | 35.14 |
| >GCSE | 35.31 | 41.55 |
| Education of partner (%) | *n=6,487* | *n=5,803* |
| <GCSE | 4.95 | 4.86 |
| GCSE | 47.43 | 47.06 |
| >GCSE | 47.62 | 48.08 |
| Housing tenure (%) | *n=12,863* | *n=7,678* |
| Council/HA rent | 16.62 | 10.25 |
| Private rent | 7.54 | 5.78 |
| Own/mortgage | 75.84 | 83.97 |
| Marital Status (%) | *n=13,289* | *n=7,939* |
| Single | 12.66 | 9.38 |
| Cohabiting | 12.53 | 9.57 |
| Married | 74.82 | 81.04 |
| Employment- mother (%) | *n=10,424* | *n=6,757* |
| Unemployed | 3.98 | 3.40 |
| Housewife/retired/education | 50.08 | 46.03 |
| Employed | 45.94 | 50.57 |
| Employment- partner (%) | *n=11,535* | *n=7,503* |
| Unemployed | 8.46 | 6.28 |
| Househusband/retired/education | 2.36 | 2.16 |
| Employed | 89.18 | 91.56 |
| Mother's age at birth, years, mean (SD) | *n=13,894* | *n=8,105* |
|  | 27.98 (4.97) | 28.95 (4.61) |
| Financial difficulties | *n=11,662* | *n=7,882* |
|  | 16.20 | 14.72 |
| Large family size | *n=11,813* | *n=7,925* |
|  | 5.95 | 4.93 |
| Gestation in weeks, mean (SD) | *n=14,422* | *n=8,105* |
|  | 38.41 (5.48) | 39.48 (1.83) |
| Male child (%) | *n=14,665* | *n=8,132* |
|  | 51.35 | 50.24 |
| Birth weight in g, mean (SD) | *n=13,716* | *n=8,013* |
|  | 3393.54 (570.73) | 3430.23 (537.32) |
| Mother reports smoking at 18 weeks pregnant (%) | *n=13,188* | *n=7,965* |
|  | 25.14 | 19.33 |

*Note: number of observations- not all participants recorded data for every characteristic. HA= housing association, GCSE= General Certificate of Secondary Education.*
