# Supplementary material for: Socioeconomic Associations with ADHD: Findings from a Mediation Analysis
Source: PLoS One. 2015 Jun 1;10(6):e0128248. doi: 10.1371/journal.pone.0128248 (PMC4451079; doi:10.1371/journal.pone.0128248)
Supplement: S3 Table — (DOCX) [file pone.0128248.s003.docx]

**Supplementary Information S3: Results of logistic regression and mediation analysis with multiple imputation for missing data**

**Table A**: Logistic regression of SES dimensions at age 0-2 on ADHD at age 7, original data (missing cases omitted from each analysis) and imputed data.

|  | **Original Data** | | **Imputed** | |
| --- | --- | --- | --- | --- |
| **Predictors** | **OR (95% CI)** | ***p*** | **OR (95% CI)** | ***p*** |
|  |  |  |  |  |
| Weekly Income (%) | N=6,698 | 0.258 | N=8,132 | 0.074 |
| >£400 | Reference |  | Reference |  |
| £300-£399 | 0.83 (0.49-1.41) |  | 0.88 (0.52-1.48) |  |
| £200-£299 | 1.02 (0.63-1.63) |  | 1.12 (0.71-1.77) |  |
| £100-£199 | 1.44 (0.87-2.39) |  | 1.61 (1.00-2.60) |  |
| <£100 | 1.46 (0.76-2.80) |  | 1.72 (0.94-3.15) |  |
| Education of mother (%) | N=7,868 | 0.491 | N=8,132 | 0.406 |
| > GCSE | Reference |  | Reference |  |
| GCSE | 1.23 (0.86-1.76) |  | 1.25 (0.88-1.79) |  |
| <GCSE | 1.19 (0.80-1.79) |  | 1.23 (0.83-1.83) |  |
| Education of partner (%) | N=5,803 | 0.168 | N=8,132 | 0.067 |
| >GCSE | Reference |  | Reference |  |
| GCSE | 1.25 (0.84-1.87) |  | 1.34 (0.93-1.92) |  |
| <GCSE | 2.01 (0.97-4.16) |  | 2.01 (1.09-3.71) |  |
| Housing tenure (%) | N=7,678 | 0.015 | N=8,132 | 0.014 |
| Own/mortgage | Reference |  | Reference |  |
| Private rent | 0.96 (0.47-1.97) |  | 0.97 (0.48-1.97) |  |
| Council/HA rent | 1.93 (1.27-2.93) |  | 1.84 (1.22-2.76) |  |
| Marital Status (%) | N=7,939 | 0.025 | N=8,132 | 0.029 |
| Married | Reference |  | Reference |  |
| Cohabiting | 1.51 (0.94-2.41) |  | 1.48 (0.92-2.40) |  |
| Single | 1.77 (1.13-2.76) |  | 1.70 (1.09-2.66) |  |
| Employment- mother (%) N=6,757 | | 0.810 | N=8,132 | 0.847 |
| Employed | Reference |  | Reference |  |
| Housewife/retired/education | 0.97 (0.60-1.37) |  | 0.99 (0.71-1.39) |  |
| Unemployed | 1.30 (0.56-3.03) |  | 1.26 (0.56-2.84) |  |
| Employment- partner (%) N=7,503 | | 0.708 | N=8,132 | 0.610 |
| Employed | Reference |  | Reference |  |
| Househusband/retired/education | 0.93 (0.29-2.96) |  | 1.01 (0.33-3.10) |  |
| Unemployed | 1.29 (0.71-2.35) |  | 1.34 (0.74-2.43) |  |
| Mothers age at birth, years, mean (SD) | N=8,105 |  | N=8,132 |  |
|  | 0.96 (0.93-0.99) | 0.016 | 0.96 (0.93-0.99) | 0.017 |
| Large family size | N=7,925 |  | N=8,132 |  |
|  | 1.64 (0.92-2.91) | 0.115 | 1.59 (0.89-2.82) | 0.115 |
| Financial difficulties | N=7,882 |  | N=8,132 |  |
|  | 2.28 (1.61-3.23) | <0.001 | 2.23 (1.57-3.16) | <0.001 |
